# Leveraging Mendelian randomization to inform drug discovery and development for ischemic stroke

**DOI:** 10.1177/0271678X241305916

**Published:** 2024-12-04

**Authors:** Iyas Daghlas, Dipender Gill

**Affiliations:** 1 Department of Neurology, University of California San Francisco, San Francisco, CA, USA; 2 Department of Epidemiology and Biostatistics, School of Public Health, Imperial College London, London, UK

**Keywords:** Drug development, drug target, ischemic stroke, Mendelian randomization, stroke

## Abstract

Discovery and development of efficacious and safe pharmacological therapies is fraught with challenges. As proteins constitute the majority of drug targets and are encoded by genes, naturally occurring genetic variation within populations can provide valuable insights to inform drug discovery and development efforts. The drug target Mendelian randomization (MR) paradigm leverages these principles to investigate the causal effects of drug targets in humans. This review examines the application of drug target MR in informing the efficacy and development of therapeutics for ischemic stroke prevention and treatment. We consider applications of MR for existing and novel treatment strategies, including targeting blood pressure, lipid metabolism, coagulation, inflammation and glycemic control. Several of these genetically supported targets are under evaluation in late-stage clinical trials. Methodological limitations of drug target MR are addressed, followed by an outline of future research directions. We anticipate that careful application of drug target MR will enhance the efficiency of drug development for ischemic stroke, consequently accelerating the delivery of effective medications to patients.

## Introduction

**Figure 1.**
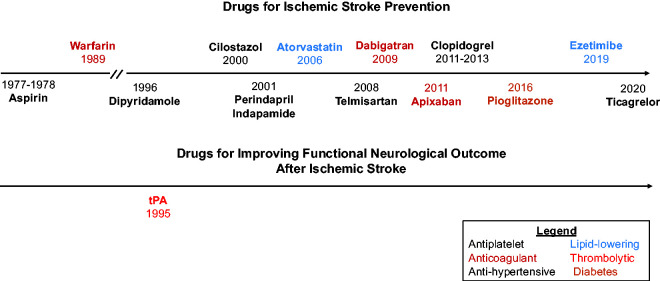
Timeline of successful phase 3 trials evaluating drugs for ischemic stroke prevention and improvement of functional outcomes. Citations for these clinical trials are as follows: aspirin,^141^ warfarin,^142^ dipyridamole,^143^ cilostazol,^144^ perindapril & indapamide,^145^ atorvastatin,^38^ telmisartan,^146^ dabigatran,^147^ apixaban,^74,148^ clopidogrel,^149,150^ pioglitazone,^110^ ezetimibe,^39^ and ticagrelor.^151^ Trials testing drugs for non-inferiority were not included in this figure (e.g., rivaroxaban and tenecteplase). tPA: tissue plasminogen activator.

Ischemic stroke is a leading cause of morbidity and mortality worldwide.^1^ The development of drugs for stroke treatment, encompassing acute care, prevention, and recovery, is a crucial research priority. Research efforts have successfully identified several preventive agents for ischemic stroke including antiplatelet, anticoagulant, anti-hypertensive, and lipid-lowering medications (Figure 1). These discoveries have contributed to advancements in stroke care and saved patient lives. Nevertheless, these drugs are imperfect, with either limited efficacy or undesirable adverse effects. Furthermore, certain stroke subtypes, such as those arising from cerebral small vessel disease, lack dedicated pharmacological interventions. Finally, thrombolytic agents remain the sole drug class demonstrating improved functional neurological outcomes following stroke, with no other pharmacological interventions showing comparable efficacy. These unmet needs warrant ongoing drug discovery and development efforts for ischemic stroke.

Drug discovery and development is fraught with major challenges at all stages.^2,3^ These challenges begin with the weak predictive value of pre-clinical data, which can lead to misguided decisions in early development.^4,5^ At the clinical trial stage, drug development efforts may be affected by suboptimal design of trials,^6^ poor recruitment and retention,^7^ and efficacy or safety issues. Throughout the process, development efforts contend with exceptionally high costs.^8^ Consequently, only fifteen percent of drugs entering phase 1 clinical trials gain regulatory approval, with development costs per approved drug ranging from $1-2 billion.^3,9^

The development of drugs for ischemic stroke is further complicated by domain-specific challenges. There are several subtypes of ischemic stroke, each characterized by distinct pathophysiological mechanisms with differential response to treatment. The most common subtyping approach was designed in the Trial of ORG 10172 in Acute Stroke Treatment (TOAST).^10^ The TOAST scheme subtypes ischemic stroke into pathophysiological mechanisms of large-artery atherosclerotic stroke (LAAS), cardioembolic stroke (CES), small-vessel stroke (SVS), ‘stroke of other determined etiology,’ and ‘stroke of undetermined etiology’.^10^ These distinct biological mechanisms often warrant different therapeutic strategies. For instance, single or dual-antiplatelet therapy is the standard of care for prevention of LAAS and SVS, whereas oral anticoagulants are the standard of care for prevention of CES due to atrial fibrillation. Unfortunately, most ischemic stroke trials lack well-powered subtyping data, leaving uncertainty about whether approved therapies show differential efficacy across stroke subtypes. Drug development for acute neuroprotection and neurorecovery is further complicated by both heterogeneous recovery patterns and the time-dependent neuronal response to ischemic injury.^11–14^ Finally, the limited translatability of preclinical data has been a major challenge for the field of stroke recovery.^15^ As a result of these limitations, no drugs apart from thrombolytics have demonstrated efficacy for improving functional neurological outcomes in phase 3 trials (Figure 1).^15^ These challenges in traditional approaches to drug discovery and development highlight the need for alternative strategies.

The analysis of human genetic variation has emerged as a tool for enhancing drug development, with genetically supported targets showing 2.6-fold higher approval rates.^16,17^ Genetic data offer several key advantages.^2^ First, the use of human data enhances the translational potential of findings as compared to data from model organisms. Second, germline genetic variants possess inherent properties that support causal inference from their association with human phenotypes, most notably that genetic variation is generally randomly allocated and thus relatively devoid of environmental confounding factors.^2,17^ Third, rigorous statistical thresholds for genetic associations and requirements for independent replication have substantially reduced rates of false positive findings.^18^ Finally, large genome-wide association study (GWAS) datasets are readily available for analysis across numerous phenotypes relevant to drug development including clinical risk factors, biomarkers, disease risk, and disease outcome.

## Mendelian randomization

### Overview

**Figure 2.**
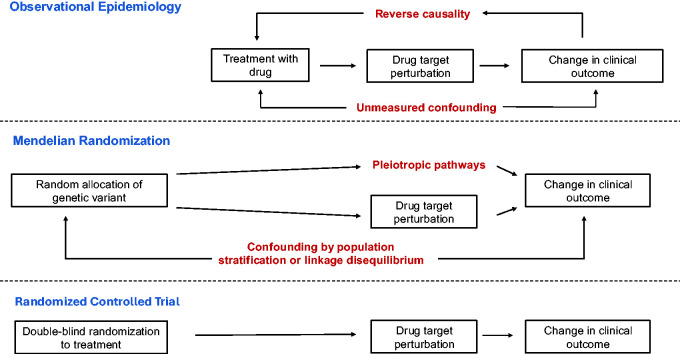
Comparison of causal inference through observational epidemiology, Mendelian randomization, and randomized controlled trials. Potential biases are displayed in red text.

The Mendelian randomization (MR) paradigm is a powerful genetic tool for inferring causal relationships between exposures and health outcomes.^2,19^ These exposures can span clinical risk factors (e.g. systolic blood pressure^20^), health behaviors (e.g. sleep patterns^21^) and alterations in protein function or abundance. MR enables causal inference by leveraging two fundamental properties of genetic variants that allow their interpretation as natural randomization experiments (Figure 2). First, germline genetic variants are generally randomly allocated during gametogenesis, mitigating their confounding by environmental factors that often limit causal inference in epidemiologic analyses. Second, the assignment of a genetic variant is typically fixed at gametogenesis and unaffected by disease status. This property mitigates bias due to reverse causality, whereby a disease outcome may influence the exposure.

MR analysis relies on several key assumptions, which can be understood by analogy to a clinical trial of blood pressure lowering for ischemic stroke prevention (Table 1 and Figure 2). First, the genetic variant must have a strong statistical association with the exposure. This is analogous to confirmation that randomization to an antihypertensive significantly lowers blood pressure in the treatment group relative to the placebo group. Second, the association of the genetic variant with the exposure must be unconfounded, akin to the assumption that covariates are balanced across treatment and placebo arms. Confounding in MR can manifest through several mechanisms, including conventional confounding by associations of environmental variables with the genetic variant, population stratification due to differences in allele frequency across ancestry groups, and linkage disequilibrium-induced confounding from correlated neighboring genetic signals (discussed later in this Review). Environmental confounding can be tested by comparing confounder distributions across variant alleles. Population stratification can be addressed through the implementation of statistical techniques, such as adjusting genetic associations for principal components of genetic ancestry.^22^ The third MR assumption is that the variant must influence the outcome solely through its effect on the exposure. This is analogous to the assumption that randomization influences the outcome through treatment with the anti-hypertensive, and not through alternative pathways such as differences in clinical practice patterns across treatment arms. Genetic proxies that do not meet this assumption are described as pleiotropic (Figure 2). Analytic strategies to mitigate bias due to pleiotropy in MR will be discussed later in this Review.

**Table 1.** Assumptions underlying causal inference in a Mendelian randomization (MR) analysis and comparison to a randomized controlled trial of blood-pressure lowering.

Assumption	MR definition	Clinical trial analogy
Relevance assumption	The genetic proxy must have a strong statistical association with the exposure	Randomization should result in a treatment group with significantly lower average blood pressure compared to the placebo group
Independence assumption	The association of the genetic proxy with the exposure is not confounded by environmental variables, genetic ancestry, or nearby genetic signals (i.e., linkage disequilibrium)	Confounders (e.g. age, socioeconomic status) are equally distributed across the treatment and placebo arm
Exclusion restriction assumption	The genetic proxy only affects the outcome through the exposure, and not through alternative pleiotropic pathways.	Randomization to the intervention arm only affects the outcome through treatment with the anti-hypertensive agent, and not through other aspects of care or patient behavior

### Drug target MR

MR may be applied to investigate potential causal effects of drug target perturbation on clinical outcomes, an approach referred to as ‘drug target MR’.^2,23^ The analytic process of drug target MR begins with selection of a drug target, which may be motivated by interest in a specific molecular pathway, evidence from preclinical studies, or opportunities to repurpose existing therapeutic agents. Alternatively, investigators may perform a hypothesis-generating survey of a wide range of potential targets.^24^ After identifying the drug target of interest, genetic variants must be carefully selected to serve as valid proxies for the exposure. Genetic variants may be prioritized according to location, functional genomic consequence, and association with biomarkers or traits mimicking drug target effect. Drug target MR generally prioritizes variants located within or near the gene encoding the target protein (*cis*-variants), as these are more likely to have specific effects on that gene’s function.^25^ Variants positioned further from the target gene (*trans*-variants) have a higher likelihood of pleiotropic effects on multiple pathways, which can invalidate the instrumental variable assumptions required for causal inference.^2^ Drug target MR may be performed irrespective of functional consequence of the sequence variant, though deleterious exonic variants^26^ are more likely to affect the gene of interest and may provide more reliable causal inference.^2^

Selected genetic variants should demonstrate association with downstream biological effects that reflect pharmacological perturbation of the target, thereby validating their use as genetic proxies in MR. In addition to clinical risk factors and outcomes, common molecular phenotypes used to proxy drug target perturbation include gene expression levels (expression quantitative trait loci, or eQTLs), circulating protein levels (protein quantitative trait loci, or pQTLs), or metabolites (metabolite quantitative trait loci, or mQTLs) that reflect target engagement. In a hypothetical example of a drug target MR analysis proxying ACE inhibition, these phenotypes could be tissue-specific genetic expression of *ACE*, circulating ACE levels, angiotensin II levels (the peptide produced when ACE cleaves angiotensin I), or systolic blood pressure.^27,28^ Concordant associations across multiple phenotypes of drug perturbation—such as a variant that both reduces circulating ACE levels and lowers systolic blood pressure—enhance confidence that the genetic variant effectively mimics drug target perturbation.

The investigator must then select appropriate study designs and outcomes that address research questions across the various phases of drug development. These may include assessment of target engagement, evaluation of potential adverse effects, optimization of clinical trial design, efficacy assessment, and exploration of repurposing potential.^2^ In most cases, genetic associations with the outcome of interest are measured in a dataset that does not overlap with the dataset used to estimate genetic associations with the exposure. This two-sample MR approach can mitigate bias from sample overlap and allows for data with the largest available sample size to be used.^29^ These associations are then aligned and harmonized with genetic associations mimicking drug target effects. Statistical methods for instrumental variable analysis are then used to estimate the MR association, which mainly serves to scale the genetic association with the outcome per unit change in a biomarker reflecting drug target perturbation.^19,30,31^

### Overview of review

Drug target MR has been increasingly applied to investigate therapeutic targets for ischemic stroke.^32^ These analyses are enabled by large GWAS of ischemic stroke, with the most recent meta-analysis (GIGASTROKE, 2002) including 110,182 stroke cases and 1,503,898 controls.^33^ A subset of these cases were classified according to TOAST criteria, permitting granular investigations of stroke pathophysiology. Prior iterations of this ischemic stroke GWAS that have been used in MR analyses include METASTROKE^34^ (2012), SiGN^35^ (2015) and MEGASTROKE^36^ (2018). GIGASTROKE and other publicly available genetics datasets for cerebrovascular traits are summarized in Table 2.

**Table 2.** Publicly available genome-wide association study datasets for ischemic stroke and cerebrovascular diseases.

Dataset category	Phenotype	Consortium or cohort	Sample size (Ancestry)
Ischemic stroke	Ischemic stroke and subtypes	GIGASTROKE^33^	110,182 cases & 1,503,898 controls (European)
	Early-onset ischemic stroke	ISGC^133^	16,730 cases & 599,237 controls (European)
Hemorrhagic stroke	Subarachnoid hemorrhage and intracranial aneurysm	Multiple cohorts^134^	10,754 cases & 306,882 controls (Combined European & East Asian)
	Intracerebral hemorrhage	ISGC^46^	3,226 cases & 3,742 controls (European)
Small vessel disease	WMH volume, fractional anisotropy, mean diffusivity	Multiple cohorts^135^	Up to 42,310 participants (European)
	Cerebral microbleeds	Multiple cohorts^136^	3,556 cases & 25,862 controls (predominantly European)
	Perivascular space burden	Multiple cohorts^137^	Up to 9,339 cases and 30,756 controls (predominantly European)
Carotid disease	Carotid intima-media thickness	Multiple cohorts^138^	116,313 participants (predominantly European)
	Carotid plaque	CHARGE, UCLEB^139^	48,434 participants (European)
Stroke recovery	Modified Rankin scale	GISCOME^120^	6,165 participants (European)
	Motor recovery	VISP trial^140^	488 participants (predominantly European)

For phenotypes with multiple available datasets, we present only the largest study. Web links are provided at the end of the manuscript. CES: cardioembolic stroke; COMPASS: Consortium of Minority Population Genome-Wide Association Studies of Stroke ISGC: international stroke genetics consortium; LAAS: large artery atherosclerotic stroke; SVD: small vessel disease; SVS: small vessel stroke; WMH: white matter hyperintensity.

In this Review we examine approved and investigational ischemic stroke drug targets that have been investigated with MR. We critically review analytic approaches and key results from these studies. We then address broad limitations of this methodology, as well as challenges specific to application of drug target MR to ischemic stroke research. Finally, we explore future directions for leveraging MR for ischemic stroke drug development.

## Lipid-lowering mechanisms

### Overview of lipid-lowering therapies for the prevention of ischemic stroke

Low-density lipoprotein (LDL) cholesterol-lowering is a core part of secondary stroke prevention.^37^ Statins, which inhibit HMG-CoA reductase, have been the mainstay of this therapeutic strategy, particularly since publication of the Stroke Prevention by Aggressive Reduction in Cholesterol Levels (SPARCL) trial (Figure 1).^38^ In SPARCL, high-dose atorvastatin therapy significantly reduced recurrent ischemic stroke risk in patients with non-cardioembolic stroke. Although statins are the most widely prescribed lipid-lowering agent, clinical trial evidence also supports PCSK9 inhibitors and ezetimibe (targeting NPC1L1) as effective for ischemic stroke prevention.^39,40^ Despite these data, there remain several uncertainties regarding the efficacy of LDL-lowering by stroke subtype, the comparative effectiveness of lipid-lowering drug classes for ischemic stroke risk reduction, and the impact of targeting non-LDL cholesterol fractions for stroke prevention. Additionally, SPARCL raised concern that statin therapy may increase risk of intracerebral hemorrhage (ICH),^38^ though findings from meta-analyses have been mixed.^41^ These outstanding questions have been investigated using MR.

### LDL cholesterol-lowering

It is uncertain whether the effect of LDL cholesterol-lowering varies by ischemic stroke subtype. This was first investigated by Hindy et al. using genetic associations with LDL from the Global Lipids Genetics Consortium (GLGC) and genetic associations with ischemic stroke from SiGN.^42^ This analysis considered LDL-lowering broadly, and so used variants from across the genome rather than restricting analysis to variants near genes encoding drug targets. MR analyses showed a protective association of LDL-lowering with risk of overall ischemic stroke and LAAS, and demonstrated null associations with risk of CES and SVS. These findings were consistent in multivariable MR analyses adjusting for genetically proxied levels of high-density lipoprotein (HDL) cholesterol and triglycerides.^42^ Multivariable MR analyses are necessary in this setting because genetic variants often have pleiotropic effects on multiple lipid subtypes.^43^ These findings have since been replicated using more recent data from the GIGASTROKE consortium.^44^ Taken together, these data suggest that the efficacy of LDL cholesterol-lowering is limited to the prevention of ischemic strokes due to an atherosclerotic mechanism.

### LDL cholesterol-lowering agents

Drug target MR has been applied to test the association of LDL cholesterol-lowering through specific drug target mechanisms.^42,44^ In these studies, genetic proxies within genes encoding drug targets (*HMGCR, PCSK9, NPC1L1*) were selected based on statistical association with LDL cholesterol levels. We focus here on analyses using the most recent data from GIGASTROKE.^44^ These analyses identified a protective association of genetically proxied NPC1L1 inhibition on risk of SVS. Further investigation of biomarkers of cerebral small vessel disease revealed that genetically proxied NPC1L1 inhibition reduced the burden of dilated perivascular spaces but had no association with white matter hyperintensity volume. Genetic proxies for PCSK9 and HMGCR inhibition were not associated with ischemic stroke or its subtypes, though wide confidence intervals do not exclude potential protective associations (odds ratio for association with LAAS [OR] 0.63, 95% confidence interval [CI] 0.31–1.26). Consequently, MR cannot yet generate compelling evidence regarding the efficacy of HMGCR inhibition for ischemic stroke prevention. This highlights the importance of considering statistical power before interpreting a null result in an MR analysis.

### Genetically proxied LDL-lowering, HMGCR inhibition, and hemorrhagic stroke risk

Falcone et al. applied MR to investigate the association of genetically proxied LDL cholesterol with ICH risk.^45^ Genetic proxies for circulating lipids were identified using data from the GLGC, and genetic associations with ICH were drawn from a 2014 GWAS meta-analysis of European ancestry cohorts.^46^ MR analyses showed an association of genetically proxied increased LDL-cholesterol with reduced ICH risk. This association was stronger for the lobar subtype of ICH. Two other MR analyses investigated the same ICH dataset using slightly different methods but did not find a significant association between genetically predicted LDL-cholesterol levels and the risk of ICH.^47,48^ More recently, an MR analysis performed using an updated GWAS of ICH also showed a null association of genetically proxied LDL-cholesterol on ICH risk.^49^ This GWAS meta-analysis included ICH cases from epidemiologic cohorts that were not radiographically validated, and so may have included alternative etiologies of intracranial hemorrhage (e.g. traumatic subdural hemorrhage) that introduced bias towards the null. Sun et al. also investigated this association using data from the China Kadoorie Biobank. The point estimate from the MR analysis was consistent with a protective association of higher LDL-cholesterol levels on ICH risk, though the confidence interval overlapped the null (odds ratio 1.13, 95% CI 0.91–1.40).^50^ Larger genetic studies of ICH and its subtypes are needed to clarify these conflicting results.

Yu et al. utilized genetic associations with lipids in the UK Biobank to identify genetic proxies for HMGCR inhibition and tested associations with ICH from the 2014 GWAS.^46,47^ Genetically proxied HMGCR inhibition was associated with increased overall and deep ICH risk, whereas associations of genetic proxies for NPC1L1 and PCSK9 with ICH were null. To further investigate this question, Mayerhofer et al. implemented a novel MR approach using the magnitude of on-statin LDL-lowering as the exposure.^51^ Genetic associations with degree of LDL-lowering were drawn from a meta-analysis of GWAS performed in epidemiologic cohorts and clinical trials of statin therapy.^52^ In a cohort of statin users in the UK Biobank, a greater magnitude of genetically predicted on-statin LDL-lowering was associated with increased ICH risk. This method was validated by demonstrating that genetically predicted on-statin LDL-lowering was associated with reduced risk of coronary artery disease and peripheral artery disease. These findings overall support the notion that statins increase ICH risk, a hypothesis that is being tested in the SATURN trial (Table 3).^53^ Despite these findings, the overall cardiovascular benefits and reduction in mortality^54^ associated with statin therapy likely outweigh the potential ICH risk.

**Table 3.** Non-exhaustive list of ischemic stroke drug targets supported by MR and currently in phase three trials.

Drug target	Drug target MR finding	Clinical trial (year of initiation)	Clinical intervention	Primary outcome
HMGCR inhibition (statins)	Increased risk of ICH	SATURN (2020)	Statin discontinuation	Recurrent ICH
IL-6 signaling inhibition	Reduced risk of overall ischemic stroke, LAAS, and SVS	ZEUS (2021)	Monthly treatment with Ziltivekimab, a novel IL-6 inhibitor	Composite CVD endpoint including ischemic stroke; ischemic stroke also included as a stand-alone secondary outcome
		IRIS-sICAS (2024)	Acute treatment with Tocilizumab, an IL-6 receptor antagonist, after stroke due to intracranial artery stenosis	Recurrent ischemic stroke within seven days
Factor XI (FXI) inhibition	Reduced risk of overall ischemic stroke and CES	LIBREXIA-STROKE (2023)	Daily treatment with Milvexian, an oral FXI inhibitor	Ischemic stroke
		OCEANIC-STROKE (2023)	Daily treatment with Asundexian, an oral FXI inhibitor	Ischemic stroke
Lp(a) lowering	Reduced risk of overall ischemic stroke and LAAS. Increased risk of SVS.	Lp(a)HORIZON (2019)	Monthly injection of Pelacarsen (antisense oligonucleotide)	Composite CVD endpoint including ischemic stroke
		ACCLAIM-Lp(a) (2024)	Subcutaneous injection of Lepodisiran (short interfering RNA)	Composite CVD endpoint including ischemic stroke; ischemic stroke also included as a stand-alone secondary outcome
		Ocean(a) (2022)	Q-3 month injection of Olpasiran (short interfering RNA)	Composite CVD endpoint that does not include ischemic stroke; ischemic stroke included as a stand-alone secondary outcome

Here, we include trials that report ischemic stroke outcomes, whether as primary or secondary endpoints. CVD: cardiovascular disease; ICH: intracerebral hemorrhage; IL-6: interleukin 6; LAS: large artery atherosclerotic stroke; SVS: small vessel stroke.

### HDL cholesterol and CETP inhibition

Hindy et al. also investigated associations of genetically proxied HDL cholesterol levels with ischemic stroke risk.^42^ Genetically proxied HDL cholesterol was associated with reduced risk of SVS, a finding that was subsequently replicated by Georgakis et al. in MEGASTROKE.^48^ Georgakis et al. also showed protective associations of genetically proxied HDL cholesterol with reduced white matter hyperintensity volume. Analyses stratified by size of HDL lipoprotein particles showed that the genetic association of HDL with SVS was driven by medium-sized HDL particles, a finding that was recently replicated using data from GIGASTROKE.^55^ These data lend support to the hypothesis that HDL-increasing therapies may confer protection against cerebral small vessel disease.

Cholesteryl ester transfer protein (CETP) inhibitors, such as anacetrapib, increase HDL cholesterol levels.^56,57^ The REVEAL trial demonstrated that treatment with anacetrapib reduced the risk of a composite cardiovascular disease endpoint, though the impact of CETP inhibition on ischemic stroke risk remains uncertain.^57^ To investigate this question, Georgakis et al. genetically proxied CETP inhibition by identifying variants within *CETP* that were associated with increased HDL cholesterol.^48^ Genetic associations with ischemic stroke were drawn from MEGASTROKE. Genetically proxied CETP inhibition was associated with reduced risk of SVS (OR 0.82, 95% CI 0.75–0.89) and reduced volume of white matter hyperintensities. However, CETP inhibition was associated with increased ICH risk (OR 1.64, 95% CI 1.26–2.13). In a subsequent analysis, Schmidt et al. investigated the diverse health effects associated with genetic variants that mimic CETP inhibition.^58^ In contrast to the prior analysis, this study identified genetic proxies for CETP inhibition as variants associated with circulating CETP levels. This approach yielded fewer genetic proxies (14 variants) relative to the HDL cholesterol-weighted approach (22 variants). MR analyses using this approach yielded non-significant protective associations with SVS (odds ratio 0.95, 95% CI 0.85–1.06). While this result was not statistically significant, the confidence intervals overlapped with the association reported in the previous study using the HDL cholesterol-weighted approach. When considering other phenotypes, genetically proxied CETP inhibition was significantly associated with increased risk of age-related macular degeneration and with reduced risk of coronary artery disease, heart failure, diabetes, and chronic kidney disease. To date, these predicted effects of CETP inhibition have not been recapitulated in clinical trials, though follow-up duration has been limited.^59^

### Triglycerides

Hindy et al. found no association between genetically proxied triglyceride levels and ischemic stroke or its subtypes.^42^ While no triglyceride-lowering agents have demonstrated efficacy in cardiovascular outcome trials, several drug mechanisms are under active investigation including inhibition of Angiopoietin-like 3 (ANGPTL3), ANGPTL4, and Apolipoprotein C3 (APOC3). Inhibiting ANGPTL3/4, and thereby enhancing lipoprotein lipase (LPL) activity, results in reduced levels of triglycerides and LDL cholesterol, and increased HDL cholesterol.^60^ Landfors et al. proxied genetic inhibition of ANGPTL3, ANGPTL4, APOC3, and LPL using *cis* variants associated with either circulating levels of the respective protein or with triglyceride levels in UK Biobank.^61^ These proxies were tested for associations with multiple cardiovascular outcomes which included overall ischemic stroke risk in GIGASTROKE. Results showed that genetically proxied inhibition of these four drug targets was not associated with overall ischemic stroke risk, in contrast to protective associations with coronary artery disease. This finding held true regardless of whether triglycerides or circulating protein levels were used as the biomarker for identifying genetic proxies. Additional work is required to determine which ischemic stroke subtypes may be prevented by targeting these drug mechanisms.

### Lipoprotein(a)

Lipoprotein(a) [Lp(a)] consists of an LDL-like particle containing apolipoprotein B100 that is covalently bound to apolipoprotein(a).^62^ While elevated Lp(a) is associated with increased risk of coronary artery disease and ischemic stroke, the causal nature of these relationships remains unclear.^62^ Given that Lp(a) levels are largely genetically determined, this phenotype presents an ideal candidate for drug target MR studies.

Larsson et al. investigated the association of genetically proxied Lp(a) levels with ischemic stroke and other cardiovascular outcomes in the UK Biobank.^63^ Genetic variants in *LPA* were selected based on their strong association with circulating Lp(a) levels in the CHD Exome+ Consortium.^64^ Genetically proxied increases in Lp(a) were associated with elevated ischemic stroke risk (4,602 cases). Positive associations were also identified for the outcomes of coronary artery disease, peripheral artery disease, abdominal aortic aneurysm, and multiple subtypes of valvular disease. The association of genetically proxied Lp(a) with stroke subtypes was separately investigated by Pan et al using data from the MEGASTROKE consortium.^65^ Using a more limited set of genetic variants proxying Lp(a), the authors identified a positive association of genetically proxied Lp(a) with LAAS, inverse association with SVS, and a null association with CES.

PCSK9 inhibitors reduce circulating Lp(a) levels in addition to their primary effect of lowering LDL cholesterol.^62,66^ De Marchis et al. leveraged MR to estimate the degree to which Lp(a) lowering explains the effect of PCSK9 inhibition on LAAS (in MEGASTROKE) and coronary artery disease.^67^ Genetic variants near *PCSK9* were selected based on associations with circulating PCSK9 levels and LDL cholesterol levels in the UK Biobank. Genetically proxied PCSK9 levels were associated with increased LAAS risk. Mediation analysis showed that only three percent of the total genetic association of PCSK9 inhibition with LAAS was potentially mediated through Lp(a) reduction, with similar results for the outcome of coronary artery disease. This significant association, which contrasts with prior null findings when proxying PCSK9 perturbation using LDL cholesterol as the biomarker,^44^ likely reflects greater statistical power when proxying PCSK9 inhibition using PCSK9 levels directly. These findings suggest that PCSK9 inhibition alone is unlikely to be a sufficient therapeutic strategy for addressing Lp(a)-related atherosclerotic risk. This lends further support to the ongoing development of Lp(a)-directed therapies. Novel therapies that inhibit Lp(a) synthesis, including antisense oligonucleotides (e.g. pelacarsen^68^) and small interfering RNA therapies (e.g. olpasiran),^69^ are undergoing evaluation in phase 3 cardiovascular outcome trials (Table 3).

### Lipids and stroke outcome

The association of circulating lipid subfractions with stroke outcome was investigated by Martín-Campos et al. Utilizing genetic proxies for lipid traits measured through nuclear magnetic resonance, they identified suggestive associations of low cholesterol in small and medium LDL with worse functional outcomes after ischemic stroke.^70^ These findings were not statistically significant after adjustment for multiple comparisons. Furthermore, analyses of stroke recovery may be influenced by collider bias (described in Limitations).

## Anticoagulation mechanisms

### Overview of anticoagulants for ischemic stroke

Oral anticoagulants have been used to prevent ischemic stroke due to atrial fibrillation since 1989^71^ (Figure 1). Warfarin was the first oral anticoagulant used in clinical practice, though its narrow therapeutic range motivated development of safer drugs including direct thrombin inhibitors (e.g., dabigatran) and factor Xa inhibitors (e.g., apixaban; Figure 1).^72,73^ This new generation of oral anticoagulants offers improved safety, efficacy, and convenience relative to warfarin.^73,74^ Despite these improvements, several unmet needs persist in this field. First, the bleeding risk of anticoagulants remains unacceptable to patients at high risk for systemic or intracranial bleeding.^75^ Second, oral anticoagulants are incompletely effective for ischemic stroke prevention. Third, these novel oral anticoagulants have only been studied in the context of CES prevention, and so it is unclear whether anticoagulation can prevent other types of ischemic stroke. These challenges have motivated the application of MR to the study of anticoagulation drug targets.

### Factor XI

Biochemical evidence suggests that coagulation factor XI (FXI) plays a greater role in pathologic thrombosis relative to hemostasis, making it an attractive drug target with potentially lower bleeding risk.^76^ In 2018, Gill et al. investigated the association between genetically proxied factor XI levels and risk of ischemic and hemorrhagic stroke.^77^ The study identified two variants near *FXI* associated with circulating FXI levels in individuals of European ancestry. Genetic associations with ischemic stroke were obtained from SiGN.^35^ MR analyses showed that genetically proxied increases in levels of circulating FXI were associated with increased risk of overall ischemic stroke, CES, and stroke of undetermined cause. The association of genetically proxied FXI levels with hemorrhagic stroke was inconclusive due to low statistical power (OR 1.81, 95% CI 0.44–7.38). Building on these results, Georgi et al. conducted a stratified, individual-level analysis using data from the UK Biobank cohort.^78^ In individuals with atrial fibrillation, genetically proxied Factor XI (FXI) inhibition demonstrated a more pronounced absolute risk reduction for ischemic stroke compared to the general population. However, the relative risk reduction was similar across both groups.

In 2023, we re-evaluated the association of genetically proxied FXI inhibition using larger genetic datasets for Factor XI (from the DeCODE cohort^79^) and ischemic stroke (GIGASTROKE), as well as a broader set of safety outcomes including lifespan and multiple bleeding outcomes with much large sample sizes.^80^ This analysis replicated the prior finding of a protective association of FXI inhibition on risk of ischemic stroke and CES (OR 0.78, 95% CI 0.72–0.84). There was no association of genetically proxied FXI inhibition with combined major and minor bleeding, gastrointestinal bleeding, ICH, aneurysmal subarachnoid hemorrhage, or cerebral microbleeds. Finally, genetically proxied FXI inhibition was associated with a longer lifespan, supporting a potential mortality benefit for this therapeutic strategy. Taken together, these analyses provide genetic support for FXI as a safe and effective drug target for preventing cardioembolic stroke. These findings do not support efficacy of this drug class for alternative stroke subtypes, a finding which offers insights for clinical trial design. Small molecule and monoclonal antibody inhibitors of FXI are under active investigation in clinical trials, which will prospectively test this therapeutic hypothesis (Table 3).

### Factor VII, factor VIII, and VWF

De Vries et al. performed a GWAS of circulating factor VII levels and used the identified genetic proxies in MR analyses for the outcome of ischemic stroke (MEGASTROKE).^81^ Both *cis* and *trans* genetic proxies revealed a significant association of genetically proxied factor VII levels with increased risk of overall ischemic stroke in MEGASTROKE. The same group separately investigated factor VIII and its carrier protein, Von Willebrand Factor (VWF), for association with ischemic stroke using MR.^82^ Only a *trans* approach for proxy identification was implemented. These analyses initially showed an association of both genetically proxied VWF and factor VIII with ischemic stroke. However, multivariable MR analyses adjusting for genetic associations with VWF eliminated the apparent link between factor VIII and ischemic stroke, suggesting VWF as the primary driver of this association. A key limitation of these analyses is the lack of stratification by stroke subtype. Furthermore, the use of *trans*-acting proxies may have resulted in selection of pleiotropic and invalid genetic proxies.

### Fibrinogen

Fibrinogen plays a crucial role in coagulation, where it is converted to fibrin as part of the final enzymatic reactions in the coagulation cascade. Circulating fibrinogen levels are associated with an increased risk of incident ischemic stroke.^83^ However, fibrinogen levels are subject to confounding as an acute phase reactant, introducing uncertainty about the causal link between fibrinogen and ischemic stroke. This relationship is further complicated by the existence of y’ fibrinogen, an alternatively spliced protein that may possess anticoagulant properties.^84^ The relationships of total fibrinogen and y’ fibrinogen with ischemic stroke risk were investigated by Maners & Gill et al.^85^ Genetic proxies were selected based on their association with y’ fibrinogen or with total fibrinogen. This approach only identified *cis* proxies for y’fibrinogen, and both *cis* and *trans* proxies for total fibrinogen. Higher levels of genetically proxied circulating y’ fibrinogen associated with reduced risk of ischemic stroke, LAAS, and CES. In contrast, higher levels of total fibrinogen were associated with increased risk of SVS and LAAS, and with reduced risk of CES. There was significant genetic overlap between genetic predictors of total and y’ fibrinogen, motivating mutually adjusted multivariable MR analyses. The protective association of genetically proxied y’ fibrinogen on ischemic stroke was similar in these adjusted analyses. In contrast, adjustment for y’ fibrinogen strengthened the association of total fibrinogen with overall ischemic stroke and LAAS, and nullified the association of total fibrinogen with CES risk (OR 0.62, 95% CI 0.30–1.28). These results support opposing effects of γ’ fibrinogen and total fibrinogen on ischemic stroke risk, underscoring the importance of multivariable MR analyses for proteins with overlapping genetic determinants.

### Hypothesis-free screens

Drug target MR has been used to perform exploratory screens of multiple coagulation factors and ischemic stroke risk. Harshfield et al. identified coagulation factors with published *cis* or *trans* genetic proxies and tested their association with ischemic stroke risk in MEGASTROKE.^36,86^ This analysis replicated several known associations, including a positive association of FXI with ischemic stroke and an inverse association of y’ fibrinogen with CES risk. A novel association was reported between genetically proxied levels of protein C and increased CES risk. The study revealed contrasting associations of factor VIII phenotypes with CES risk: genetically proxied increases in factor VIII activity were associated with reduced risk, while elevated factor VIII antigen levels were associated with increased CES risk.^86^ These results are challenging to reconcile with each other and with prior MR analyses of factor VIII and ischemic stroke.^82^ These opposing results are likely due to significant bias introduced by including highly pleiotropic, *trans*-acting genetic variants. For instance, the rs529565 variant in the *ABO* gene (chromosome 9) was used to proxy these factor VIII phenotypes. Variants in *ABO* have dozens of pleiotropic associations with clinically relevant traits including blood pressure, LDL cholesterol, and multiple coagulation factors, and therefore should not be used in MR analyses as genetic proxies.^86^

Yuan et al. screened coagulation factor genes for *cis*-acting genetic proxies that were significantly associated with prothrombin time (PT) or partial thromboplastin time (PTT).^87^ These variants were tested for association with ischemic stroke and subtypes using MEGASTROKE. Significant associations with overall ischemic stroke were identified for fibrinogen alpha chain (FGA) and fibrinogen gamma chain (FGG). Associations with stroke subtypes meeting study-level significance were identified for FGA (LAAS and CES), FGG (LAAS and CES), and FXI (CES). A limitation of this analysis is the mismatch between the GWAS population for the exposure phenotype (East Asian ancestry) and the GWAS population for the outcome phenotype (European ancestry).^87^ Furthermore, PT/PTT are poor measures of thrombotic liability and thus under-ascertain coagulation factor proxies for use in MR analysis.^87^

## Anti-hypertensive mechanisms

### Overview of blood pressure reduction for ischemic stroke

Clinical trials support blood-pressure reduction as an effective strategy for lowering ischemic stroke risk.^88^ There is little data as to whether this effect varies by stroke subtype, anti-hypertensive drug class, or the interaction between the two. More granular data would enable anti-hypertensive strategies tailored to specific cerebrovascular risk profiles.

### Genetically proxied anti-hypertensive drug targets and stroke risk

Gill et al. identified genetic proxies for multiple drug classes of anti-hypertensives, including beta blockers, calcium channel blockers, and ACE inhibitors.^27,89^ These variants were selected based on their association with systolic blood pressure and their proximity to genes encoding protein targets of these drugs (e.g. *ACE*). Genetic associations with stroke were drawn from MEGASTROKE. MR analyses using genetic proxies for ACE inhibition and calcium channel blockade showed protective associations with overall ischemic stroke risk. In contrast, genetically proxied beta blocker effects showed no significant association with ischemic stroke, in keeping with meta-analyses of randomized controlled trials.^90^ These genetic proxies were subsequently used by Georgakis et al. in an investigation of ischemic stroke subtypes.^91^ MR analysis was restricted to proxy effects of calcium channel blockers and beta blockers due to statistical power limitations. Genetically proxied beta blocker effects showed no significant association with ischemic stroke or its subtypes. In contrast, genetically proxied calcium channel blockade demonstrated a protective association with all ischemic stroke subtypes, and particularly pronounced associations SVS. Genetic proxies for calcium channel blockade also associated with reduced white matter hyperintensity volume, a biomarker of cerebral small vessel disease. These data suggest that calcium channel blockade may slow progression of cerebral small vessel disease, a condition with no specific treatments.

### Genetically proxied anti-hypertensive targets and functional outcomes after stroke

A similar approach was taken to investigate the association of genetically proxied anti-hypertensive drug targets on functional outcome after ischemic stroke (Table 2).^92^ This study expanded the set of proxied anti-hypertensives to also include thiazide diuretics and angiotensin receptor blockers (ARBs). Genetically proxied ACE inhibition and calcium channel blockade were both associated with better functional outcomes after ischemic stroke. No significant associations were identified for other drug targets. These results should be interpreted cautiously, as collider bias is particularly relevant to blood pressure given its strong association with ischemic stroke risk.^93^ Moreover, the genetic proxy for ARBs was in *PPARG*, which encodes a protein involved in multiple cardiometabolic pathways distinct from the primary mechanism of ARB drugs^94^; PPARG induction is a secondary effect limited to a subset of ARBs.

## Anti-inflammatory mechanisms

### Overview of inflammation and stroke

Inflammation plays a key role in ischemic stroke pathogenesis, as evidenced by findings from basic science, translational research, and epidemiology.^95–98^ Despite these consistent findings, there are no anti-inflammatory agents with proven efficacy for preventing ischemic stroke or improving post-stroke recovery.^95^ This evidence gap has motivated MR studies designed to predict the effect of perturbing inflammation-related drug targets on ischemic stroke risk.

### Interleukin-6 signaling

Interleukin 6 (IL-6) is a proinflammatory cytokine implicated in the pathogenesis of atherosclerosis.^97^ IL-6 signaling promotes hepatic synthesis of acute-phase reactants like C-reactive protein (CRP) and fibrinogen, which can serve as biomarkers of this pathway. Georgakis et al. proxied downregulated IL-6 signaling by identifying seven genetic variants *cis* to the IL-6 receptor (*IL6R*) gene that were associated with lower CRP levels.^99^ These proxies were further proven to mimic downregulation of IL-6 signaling by demonstrating an association with reduced fibrinogen levels. In MR analyses, genetically proxied downregulated IL-6 signaling was associated with reduced risk of overall ischemic stroke, LAAS, and SVS in MEGASTROKE. Protective associations were also identified for coronary artery disease, abdominal aortic aneurysm, atrial fibrillation, and carotid plaque. In a separate investigation, genetically proxied inhibition of IL-6 signaling further associated with reduced risk of ‘stroke of undetermined source’.^100^ These findings implicate IL-6 signaling in the pathogenesis of multiple stroke subtypes and cardiovascular disease, a hypothesis that is under active investigation in phase 3 clinical trials (Table 3).

### Other interleukins and cytokines

Georgakis et al. examined genetically proxied levels of 41 cytokines and growth factors with ischemic stroke risk.^101^ Both *trans* and *cis* genetic proxies for circulating cytokine levels were obtained from a study of 8,293 healthy Finnish adults, and genetic associations with ischemic stroke were obtained from MEGASTROKE. After adjusting for multiple comparisons, monocyte chemoattractant protein 1 (MCP1/CCL2) showed a significant association with increased risk of overall ischemic stroke, CES, and LAAS. This finding aligns with experimental work supporting a role for MCP-1 in the pathogenesis of atherosclerosis.^101^ However, the use of *trans* variants may have introduced bias and therefore warrants re-evaluation of this relationship with *cis* variants in future analyses.

Yuan et al. reported a suggestive association between genetically proxied interleukin-1 receptor antagonist (IL-1ra) and CES, though the association of IL-1ra with overall ischemic stroke risk and other subtypes was null.^102,103^ In a separate investigation, Yuan et al. examined the association of *trans* proxies for circulating tumor necrosis factor alpha (TNF-α) levels with stroke risk.^104^ MR analyses demonstrated positive associations with overall ischemic stroke risk in MEGASTROKE. These results warrant cautious interpretation due to the use of trans proxies positioned in genes unrelated to TNF signaling. For example, rs3184504 is a well-known missense variant in *SH2B3* that is associated with hundreds of traits at genome-wide significance.^105^ A subsequent MR analysis utilized a more biologically informed approach that identified genetic proxies for TNF-a signaling as *cis* variants near *TNFRSF1A* (encoding TNF receptor 1) that were associated with lower levels of CRP.^106^ This approach did not reveal significant associations with ischemic stroke risk in MEGASTROKE, though CIs were wide (OR 0.77, 95% CI 0.36–1.65). The relationship between genetic variation in TNF-α and stroke risk thus remains uncertain.

Drug target MR analyses have examined the association of inflammatory proteins with stroke outcome. Liu et al. used the same Finnish cytokine GWAS to test the association of genetically proxied cytokine levels on functional outcome after stroke using the GISCOME dataset (Table 2).^107^ Poor functional outcome was defined as a modified rankin scale (mRS) score of 3–6 at 60–190 days, and good functional outcome was defined as an mRS of 0–2. Genetically proxied CCL11 was associated with increased odds of a poor functional outcome after stroke. Interpretation of these findings is limited by the use of a less stringent statistical significance threshold of 5 × 10^−6^ for variant selection. Furthermore, all proxies were *trans*-acting, including several clearly pleiotropic variants (e.g., rs2229593 that influences MCP-1 levels). In a separate investigation, Wang et al. examined the association of *cis* proxies for soluble adhesion molecules with functional outcome after stroke.^108^ Significant positive associations were identified for sICAM-1 and sE-selectin. While the use of *cis* proxies is a relative strength, the statistical significance threshold for associations in variant selection *(P < *10^−4^) may have introduced weak instrument bias.

## Anti-diabetes mechanisms

### Overview

Diabetes mellitus and hyperglycemia are independently associated with risk of incident ischemic stroke and with poor functional outcomes after stroke.^109^ There is evidence that treatment with certain diabetes medications, such as thiazolidinediones^110^ and GLP-1 agonists,^111^ may mitigate these risks. Relative to other drug classes, MR has been less frequently applied to investigate anti-diabetes drug targets, primarily due to a lack of suitable genetic proxies. However, recent large-scale GWAS of diabetes and related traits have created new opportunities to apply MR in this domain.^112^

### Stroke prevention

Zhu et al. investigated the association of genetic proxies for targets of sulfonylureas, insulin analogues, GLP-1 agonists (also approved for weight loss), and thiazolidinediones on ischemic stroke risk.^113^ Variants were selected according to associations with blood glucose in non-diabetic participants in the UK Biobank. Genetic proxies for sulfonylurea targets in *KCNJ11* and *ABCC8* were strongly associated with reduced ischemic stroke risk. Other proxied drug targets did not associate with stroke. These results should be interpreted cautiously. The reported effect sizes for the association of genetically proxied sulfonylurea effects with ischemic stroke risk seem implausibly large (OR 0.06 and 0.26). Furthermore, confidence intervals for all null findings were wide (e.g., *GLP1R*: 95% CI 0.29–14) and thus do not rule out an effect of the drug mechanism on stroke risk. Concluding a null effect of GLP1R agonism would directly contradict meta-analyses of clinical trials that have shown a beneficial effect of GLP1R agonists on ischemic stroke risk.^111^

Karhunen et al. investigated the association of genetically proxied glucose-dependent insulinotropic polypeptide (GIP) signaling on various cardiometabolic outcomes including ischemic stroke (MEGASTROKE).^114^ Genetic proxies in the *GIP* and *GIPR* genes were identified based on statistical associations with type 2 diabetes (*p < *5 × 10^−6^) and concordant associations with glycated hemoglobin (*p < *0.05). MR analyses were performed only when statistical colocalization between exposure and outcome traits in the respective gene regions was evident. Despite an association of genetically proxied *GIP/GIPR* agonism with a favorable cardiometabolic risk profile (including higher BMI and HDL cholesterol), there was no strong evidence of association with ischemic stroke risk.

### Functional outcome after ischemic stroke

Observational studies suggest that metformin use prior to ischemic stroke may improve functional outcomes.^115,116^ This hypothesis has motivated MR analyses to assess causality. However, conventional MR methods, which typically investigate one molecular target at a time, face challenges with pleiotropic drugs like metformin that influence multiple biological pathways. In these instances, MR can proxy the effects of perturbing the key downstream protein targets. MR analyses of metformin have primarily focused on AMP-activated protein kinase (AMPK), which is thought to mediate a significant proportion of the downstream effects of metformin.^117,118^ Unfortunately, some studies employing this approach have incorrectly presented their findings as representative of metformin’s complete effects, rather than reflecting the component attributable to AMPK modulation.^119^

Wang et al. used this analytic framework to investigate the association of genetically proxied AMPK activation with functional outcomes after ischemic stroke.^120^ Genetic proxies were identified as variants near genes encoding AMPK subunits and that were associated with glycated hemoglobin at *p < *0.05. Their analysis showed an association between genetically proxied AMPK activation and increased odds of a good functional outcome after ischemic stroke. However, the odds ratio was 0.06, an implausibly large estimate. Another major limitation of this analysis is the use of weak genetic proxies, which can introduce significant bias in MR.^121^ Finally, demonstration of additional associations with phenotypes putatively related to AMPK activation would increase confidence in the validity of this genetic proxy

## Novel mechanisms

Drug target Mendelian randomization (MR) may be applied in a hypothesis-free approach to scan the proteome for novel associations between protein levels and clinical outcomes. This exploratory study design was implemented in 2019 by Chong et al. who assessed genetically proxied levels of 653 circulating proteins with risk of ischemic stroke subtypes in MEGASTROKE.^122^ Genetic associations with circulating protein levels were drawn from five publicly available GWAS of protein levels, including the INTERVAL study. Significant associations with stroke were identified for: CD40 (inverse association with LAAS), LP(a) (positive association with LAAS), factor XI (positive association with CES), ABO (positive associations with LAAS and CES), TNFSF12 (inverse associations with CES), MMP12 (inverse association with LAAS), and SCARA5 (inverse association with CES). Of these, genetically proxied levels of MMP12 and TNFSF12 increased risk of hemorrhagic stroke subtypes, whereas SCARA5 was inversely associated with subarachnoid hemorrhage risk. A phenome-wide MR analysis did not reveal any on-target adverse effects of SCARA5 inhibition. These data suggest that interventions aimed at enhancing activity or levels of SCARA5 may reduce risk of ischemic and hemorrhagic cerebrovascular disease. Future studies should focus on identifying the relevant molecular mechanisms and exploring the feasibility of therapeutic strategies targeting SCARA5.

In 2020, Chen et al. reported results from a similar analysis utilizing an updated iteration of the INTERVAL proteogenomic study.^123^ We summarize here results from analyses performed using *cis* genetic proxies. This analysis replicated several associations from the prior Chong et al. study for CD40 and MMP12. Novel associations were identified for TFPI (inverse association with ischemic stroke and nominal inverse association with LAAS), IL6RA (inverse association with ischemic stroke and SVS), TMPRSS5 (positive association with ischemic stroke and CES), and CD6 (positive association with ischemic stroke). MR mediation analysis was applied to explore whether these protein associations were mediated by conventional cerebrovascular risk factors. These analyses revealed that atrial fibrillation may mediate a quarter of the association of TMPRSS5 and IL6RA with ischemic stroke.

**Figure 3.**
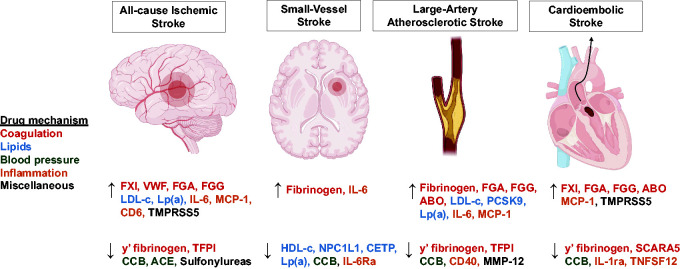
Graphical summary of ischemic stroke drug targets with supporting evidence from drug target MR. The ↑ arrow corresponds to targets where increased protein levels and/or signaling correspond to an increase in risk of the outcome. The ↓ corresponds to targets where increased protein levels and/or signaling correspond to a reduction in risk of the outcome. The exceptions to this are the blood pressure traits CCB and ACE, which both reflect the effect of inhibiting those pathways on ischemic stroke risk. The association of genetically proxied PCSK9 inhibition with LAAS was only significant when using PCSK9 levels as the exposure phenotype (in contrast to LDL cholesterol). ACE: angiotensin-converting enzyme; CCB: calcium channel blocker; CETP: cholesteryl ester transfer protein; HDL-c: high-density lipoprotein cholesterol; IL: interleukin; LDL-c: low-density lipoprotein cholesterol; Lp(a): lipoprotein(a); FGA: fibrinogen alpha chain; FGG: fibrinogen gamma chain; FXI: Factor XI; MCP-1: monocyte chemoattractant protein 1; MMP: matrix metalloproteinase; NPC1L1: Niemann–Pick C1-like 1; PCSK9: proprotein convertase subtilisin/kexin type 9; SCARA5: Scavenger Receptor Class A Member 5; TFPI: tissue factor pathway inhibitor; TMPRSS5: Transmembrane Serine Protease 5; TNFSF: Tumor necrosis factor ligand superfamily; VWF: Von Willebrand factor.

A summary of targets with Mendelian randomization evidence in ischemic stroke is provided in Figure 3.

## Limitations of drug target MR

Accurately interpreting findings from drug target Mendelian randomization (MR) analyses requires a nuanced understanding of both the general methodological limitations of the approach and those limitations specific to stroke research. Foremost among these limitations is the potential for pleiotropy and genetic confounding to invalidate genetic proxies for drug target perturbation. While it is impossible to rule out this prevalent^124^ bias, investigators can employ several strategies to mitigate its impact.^2^ First, *cis* rather than *trans-*acting variants may be prioritized for use as genetic proxies for drug target perturbation. Second, analysts may implement model-based statistical sensitivity analyses that are robust to various forms of pleiotropy.^125^ Third, databases of genotype-phenotype associations^126^ may be leveraged to screen for pleiotropic effects of genetic proxies. Such an approach clearly demonstrates that *ABO* variants are widely pleiotropic and therefore cannot be used as valid genetic proxies for coagulation factors; despite this knowledge, these variants continue to be used in drug target MR analyses. Finally, genetic confounding by linkage disequilibrium may be addressed using Bayesian colocalization, a statistical technique that account for the correlation structure between variants.^127^

A fundamental constraint of MR is the absence of suitable genetic proxies for certain drug targets. This limitation often stems from the absence of genetic variation within a specific gene that influences a biomarker of drug target perturbation. For example, large blood pressure GWAS have not identified genetic variants in genes that encode for targets of ARBs (*AGTR1/2*).^27^ In other cases, this limitation may stem from insufficient or absent genetic data for phenotypes reflecting drug perturbation. For example, platelet function assays have not been investigated in GWAS, and studies on platelet reactivity have been limited to relatively small samples.^128^ This has likely contributed to the lack of available genetic proxies that robustly mimic effects of antiplatelet drugs such as aspirin or clopidogrel. These limitations may be addressed as genetic datasets expand, encompass a wider range of phenotypes, and include participants from diverse ancestry groups.

Several additional limitations warrant consideration. First, MR analyses measure the associations of lifelong genetically predicted perturbations of drug targets. This contrasts with effects of short-term pharmacological interventions at a specific time point in the life course. The consequence of this differential exposure duration is illustrated by the example of LDL cholesterol lowering and coronary disease, where MR analyses consistently estimate larger effect sizes than in clinical trials.^129^ These differential effects are further influenced by canalization, wherein developmental compensatory mechanisms alter the impact of the variant on the outcome. Second, conventional MR analyses assume a linear relationship between the exposure and outcome, limiting their applicability to non-linear biological processes. This limitation may be addressed by using variants with large effect sizes on the exposure (e.g. loss-of-function variants) or through application of valid statistical methods for non-linear MR using individual-level data.^130^ Third, drug target *cis-*MR analyses are often underpowered due to the limited number of independent variants available at a given genetic locus. Fourth, drug target MR is typically unable to delineate the tissue or cell types through which the genetic variant influences the outcome. Incorporating datasets of genetic associations with gene expression and protein levels across multiple tissues may partially address this limitation, though definitive mechanistic insights require experimental studies. Finally, MR analyses are often restricted to European ancestry groups due to data availability, potentially reducing the generalizability of findings to other populations.

There are additional considerations for the interpretation of drug target MR analyses for ischemic stroke. There are often multiple, partially overlapping MR analyses investigating the same research question and sometimes arriving at different conclusions. In general, analyses using the most recent GWAS of stroke (e.g., GIGASTROKE) should be prioritized, as these larger sample sizes are expected to yield more reliable statistical associations. However, the overall quality of an analysis should also be considered foremost, with particular attention to the methodology used for selecting genetic proxies. A high-quality analysis using a slightly older dataset may be preferable to more recent analyses using invalid genetic proxies. An important consideration for MR analyses of stroke recovery is the possibility of collider bias.^131^ Stroke recovery cohorts are, by definition, conditioned on stroke incidence. This can induce correlations relationships between exposures for stroke incidence which in turn can create spurious ‘paths’ to the outcome of stroke recovery. Addressing this bias requires the application of specialized statistical approaches.^132^

## Future directions

Future GWAS should target clinically relevant stroke phenotypes that have not yet been sufficiently examined in genetic studies. These include genetic studies of carotid plaque, embolic stroke of undetermined source, intracranial atherosclerosis, infarct volume, hemorrhagic transformation after thrombolysis, and recurrent ischemic stroke. Similarly, larger GWAS for exposure phenotypes, such as platelet function, would enable the identification of genetic proxies for drugs that have not yet been studied using MR. As exome-sequencing datasets become more widely available, there will be opportunities to identify more genetic proxies and to perform analyses using rare, large-effect variants in MR.

Selection of valid genetic proxies is the cornerstone of causal inference in drug target MR, necessitating ongoing research to refine optimal approaches. Developing and implementing these methods will require collaboration across clinical, methodological, and translational domains. The continued development of statistical techniques to address collider bias will be essential to ensure more reliable causal inferences in analyses of stroke outcomes. Finally, developing novel strategies to mitigate pleiotropy in the context of drug target MR will continue to enhance the reliability of findings.

## Conclusion

As genetic data continue to expand, MR is poised to play an increasingly important role in identifying and validating therapeutic targets for ischemic stroke. However, to fully realize the potential of these insights, it is crucial for the drug development community to understand these genetic approaches and embrace them to improve efficiency in the research pipeline. We are optimistic that the success of human genetics in drug discovery for other conditions bodes well for its contributions to ischemic stroke research.

## Data links

Most of the cited GWAS datasets in Table 2 are available for download at the following portal: https://cd.hugeamp.org/downloads.html.
